# Preparation of PLGA-chitosan based nanocarriers for enhancing antibacterial effect of ciprofloxacin in root canal infection

**DOI:** 10.1080/10717544.2019.1701140

**Published:** 2019-12-13

**Authors:** Mona G. Arafa, Hadeel A. Mousa, Nagia N. Afifi

**Affiliations:** a Department of Pharmaceutics and Pharmaceutical Technology, Faculty of Pharmacy, The British University in Egypt (BUE), El Sherouk City, Egypt;; b Chemotherapeutic Unit, Mansoura University Hospitals, Mansoura, Egypt;; c Department of Medical Science, Faculty of Dentistry, The British University in Egypt (BUE), El Sherouk City, Egypt;; d Department of Pharmaceutics and Industrial Pharmacy, Faculty of Pharmacy, Cairo University, Cairo, Egypt;; e Department of Pharmaceutics and Industrial Pharmacy, Faculty of Pharmacy, October 6th University, Cairo, Egypt

**Keywords:** PLGA, chitosan, nanocarriers, ciprofloxacin, root canal

## Abstract

The aim of this study is to prepare and evaluate the antibacterial and antibiofilm activity of ciprofloxacin (CIP) loaded PLGA nanoparticles (F2) and CIP-PLGA nanoparticles coated with chitosan (F3) versus ciprofloxacin solution (Fl) as a control on *Enterococcus faecalis*. F2 was prepared using double emulsion evaporation technique then coated with chitosan (F3). The prepared F2 and F3 were evaluated for size, surface charge, encapsulation efficiency, morphology and *in vitro* release. F1, F2, F3, and Chitosan (CS) were assessed *in vitro* using agar diffusion technique and biofilm inhibition assay. Finally, biofilm inhibition on teeth using Colony Forming Unit (CFU) was implemented with different concentrations of the three formulae. The results revealed that F2 is 202.9 nm with a negative charge −0.0254 mv, while F3 is 339.6 nm with a positive charge +28.5 mv. The encapsulation efficiency of F2, and F3 was 64% and 78% respectively. The amount released was 92.62% and 78.3% for F2 and F3, respectively, after 72 h, while F1 showed 100% released in the first hour. CS, F1, F2, and F3, showed antibacterial effect with inhibition zone of 12 mm, 22 mm, 20 mm, and 32 mm respectively. Biofilm inhibition of F1, F2, and F3 were 60%, 74%, and 91.8%, respectively. F3 colony count was less than F2, and F1 in all concentrations. It can be concluded that F3 had proven to exhibit potential antibacterial and antibiofilm activity in a controlled release pattern consequently, they can be used as an intra-canal medication.

## 1. Introduction

Failure of root canal treatment is mainly attributed to eradication of bacteria and incomplete disinfection of the complex root canal system, which will inevitably lead to persistent apical periodontitis (Tabassum & Khan, 2016). *Enterococcus faecalis (E. faecalis)*, is a Gram-positive bacterium often isolated from the root-filled teeth with chronic apical periodontitis. *Enterococcus faecalis* invades the dentinal tubules, adheres to root canal wall and forms a biofilm on dentin. *Enterococcus faecalis* has the ability to deeply penetrate the dentinal tubules to a depth of 200–1500 µm, as a result; complete eradication of *E. faecalis* is quite difficult since it is inaccessible for conventional medicaments including systemic antibiotics such as; ciprofloxacin (CIP), metronidazole and sealers (Love 2001; George et al., 2005; Segura-Egea et al. 2017). Dentinal tubules canals have an average diameter ranging between 1 and 2.5 µm, (Arends et al., 1995; Wilczewska et al., 2012) while the branches have an average diameter of 0.5–1 µm (Mjör & Nordahl, 1996). The biofilm of the bacteria allows it to survive even in nutrient-depleted environment such as root-filled teeth, thus shows higher antimicrobial resistance due to the protective barrier provided by the biofilm (Dunne et al., 1993). Therefore, an antimicrobial agent that can penetrate the biofilm and eliminate the bacteria. *E. faecalis* is needed, since *E. faecalis* survives in the presence of several intra canal medications such as sodium hypochlorite, clindamycin and the most popular medication is calcium hydroxide (Camps & Pashley, 2000; Haapasalo et al., 2000). Although, triple antibiotic pastes were used to invade the biofilm and root canal infection, on the other hand, their main disadvantage was found to make teeth discoloration (Vijayaraghavan et al., 2012). Consequently, double antibiotic pastes are currently used in the root canal treatment to eliminate disadvantage of teeth discoloration, however the concentration achieved through diffusion of the antibiotics from the paste is insufficient to inhibit bacterial biofilm and may cause resistant bacteria (Athanassiadis et al., 2007; Vijayaraghavan et al., 2012). Also, silver nanoparticles were prepared to invade biofilm and they were quite effective (Halkai et al., 2018), however, toxicity is one of its major disadvantages (Swathy, 2014). In this light, there is a growing need for nanoparticles capable of delivering the antibiotic within the dentin tubules and provide a sustained drug release that has efficient antibiotic effect to invade biofilm (Saber & El-Hady, 2012). In this sight, encapsulating the suitable antibiotic ciprofloxacin in biodegradable polymeric nanoparticles will enable the drug to be delivered deep within the dentin tubules and eradicate biofilm. The ability of polymeric nanoparticles to provide sustained drug release will also allow the delivery of high concentrations of drugs over a long period (Singh & Lillard, 2009), and hence addressing the outcome of the antibiotics pastes that are in current use. Biodegradable PLGA nanoparticles have higher penetration ability through cell membranes, which is especially important in increasing antimicrobial activity against biofilm (Windley et al., 2005; Hajipour et al., 2012). On the other hand, using of chitosan which is a natural derivative known to have a great impact in endodontics due to its bio-adhesion, biodegradability, biocompatibility, antimicrobial with a low toxic profile (Kean & Thanou, 2010), also it is quite unique bio-based polymer to be used as an alternative antimicrobial agent in dentistry. Chitosan nanoparticles have been used as carriers to enhance drugs penetration efficiency (Csaba & Alonso, 2014; Rakesh Kumar Marwaha, 2014), and also to increase their antibacterial effect (Ibrahim et al., 2015) consequently chitosan was chosen to coat PLGA nanoparticles. Thus, researchers focused on nanosized which was proven to have better antimicrobial effect compared to conventional non-effective irrigating systems (Sireesha et al., 2017). This study aims to provide biodegradable nanoparticles with sustained release of the drug which can penetrate dentinal tubules and eradicate biofilm using antibacterial and antibiofilm chitosan.

## 2. Materials and methods

### 2.1 Materials

Ciprofloxacin hydrochloride was supplied by EPICO Company, Cairo, Egypt. Poly (d,l lactide-co-glycolide) ‘PLGA’ lactide: glycolide 50:50 ester terminated molecular weight 38,000–54,000 Da, chitosan low molecular weight 50,000–190,000 Da and Poly vinyl alcohol molecular weight 31,000–50,000 were supplied by Sigma Aldrich, Berlin, Germany. Brain heart infusion broth was supplied by LAB M United Kingdom, Crystal Violet supplied by Oxford Lab Chem, Mumbai, India, Tryptone soya broth was supplied by OXOID, England. Cellophane membrane 12–14 kDa supplied by Spectrum Medical Inc, Raleigh, NC. Normal saline for IV injection and Glucose 5% for IV injection supplied by Nasr Company, Cairo, Egypt. Ethanol was supplied by Fisher Scientific, Loughborough, UK. Hydrochloric acid was supplied by Al Ahram Laboratory Chemicals CO, Cairo, Egypt Dichloromethane and Acetic acid were supplied by Pio Chem, Cairo, Egypt. Human extracted single rooted teeth provided from dentistry hospital of the British University in Egypt.

### 2.2. Nanoparticles preparation

PLGA nanoparticles containing CIP were prepared using double emulsion solvent evaporation technique. Briefly 50 mg of CIP were dissolved in 2 ml 1%PVA solution and sonicated using probe sonicator (Sonics Vibra Cell power 130 watt, frequency 20 kHz, made by Sonics & Materials Inc, Newtown, CT) for 30 s, 50 amplitude to form the aqueous phase (w_1_). Two hundred and fifty mg of PLGA were dissolved in 5 ml dichloromethane to form oil phase (o) (Dillen et al., 2004). Primary emulsion was formed by adding w_1_ to oil phase then sonicated at 80 amplitude for 3 minutes. Secondary emulsion was prepared by adding the primary emulsion to 25 ml 1% PVA with and without chitosan, the amount of chitosan added is listed in (Table 1) (Pandit et al., 2017) and sonicated for 3 min at 80 amplitude, then homogenized using (wisetis homogenizer 280 W 50/60 Hz, made by DAI Hau Scientific & Co, Seoul, Korea) at 8000 rpm for 15 min, finally it was sonicated for 30 min at 80 amplitude (Dillen et al., 2004; Mir et al., 2017). To prevent coalescence of the secondary emulsion, it was added to 60 ml 0.5%PVA (Dillen et al., 2004). The formed nanoparticles were left on the stirrer (Accuplate Hot plate stirrer, Lab Net International, Mexico) for 1–2 h at 1000 rpm in room temperature (Parveen et al., 2012; Mustafa et al., 2017). Nanoparticles were collected by centrifugation at 12,000 rpm at 4 °C for 20 min, washed then kept in the refrigerator for further investigations.

**Table 1.** Composition of CIP-PLGA nanoparticles coated with chitosan.

Formula	CIP/mg	PLGA/mg	Chitosan/mg
F2	50	250	0
F3	50	250	50
F4	50	250	100
F5	50	250	150
F6	50	250	200

*F1 = 50 mg ciprofloxacin solution.

### 2.3. Determination of particle size and zeta potential

Particle size was measured using (Malvern Zetasizer Instruments by Malvern, UK). Freshly prepared suspension of all formulas was measured after dilution with distilled water. Each sample was measured 3 times and the average was taken at 25 °C. Zeta potential of the formed nanoparticles was also measured to give an information on the surface charge using (Malvern Zetasizer Instruments by Malvern, UK). The freshly prepared sample was diluted 100 times and injected in the zeta particle sizer capillary cell with electrodes at both ends. All readings were made at 25 °C (Arafa & Ayoub, 2018).

### 2.4. Determination of drug encapsulation efficiency

To determine drug encapsulation efficiency percentage (EE %), the concentration of free drug in the supernatant at 276 nm using (UV spectrophotometer Jasco v-630, made by Jasco, Tokyo, Japan) were measured for the formulas that showed suitable size. Nanoparticles were centrifuged at 10,000 rpm for 20 min then the amount of free drug was determined. The (EE%) of the drug was determined using the following equation 1: (EE%) = {(W_initial drug_ – W_free drug_)/W_initial drug_} × 100% (Holm et al., 2001).

### 2.5. Differential scanning calorimetry (DSC) analysis

Thermal behaviors were determined for PLGA, CS, CIP, F2, and F3 (DSC Q2000, TA Instruments, made by Zellik, Belgium). Samples between 1 and 3 mg were weighed in a T zero aluminum pan sealed with an aluminum lid. They were heated from 20 °C to 400 °C using a heating rate of 10 °C/min (Sobhani et al., 2017).

### 2.6. X-ray diffraction (XRD)

A characteristic X-ray pattern obtained for each crystalline and amorphous phase present in a material used. The XRD analyses performed on PLGA, CS, CIP, F2, and F3 nanoparticles were subjected to X-ray diffraction (X’pert Pro, made by Panalytical, Netherland), equipped with a PW R30 X-ray generator. Nickel filtered Cu kα1 radiation having a wavelength of 1.5106 Å, operating at 35 KW and 20 mA in the range (2*θ*) of 5–70 degrees was used. X-ray diffracto grams were obtained at a scanning rate of 1° (2*θ*) per min (Abo-Elseoud et al., 2018).

### 2.7. Fourier transforms infrared (FTIR) spectroscopy

To determine if the drug retained its chemical characteristic peaks after encapsulation, the Fourier transform IR spectra were documented for PLGA, CS, CIP, F2, and F3. Dry potassium bromide powder was compressed with the tested samples into a disc using a hydrostatic press. The scanning range was 400–4000 cm^−1^ using (JASCO FTIR-6200, JASCO International Co., Ltd., Tokyo, Japan) (Tom et al., 2004).

### 2.8. Scanning and transmission electron microscope

The morphology and the nanostructure of the particles formulated were analyzed by scanning electron microscope (SEM) (vp supra 55 made by Zeiss, Berlin, Germany) and transmission electron microscope (TEM) (JEM made by JEOL Ltd, Peabody, MA) for F2 and F3 (Ram et al., 2018).

### 2.9. *In vitro* release study

The *in vitro* release of the drug from the nanoparticles was determined using the membrane diffusion technique (Arafa et al., 2018), using 12–14 kDa cellophane membrane, with added amounts of F2 and F3 equivalent to 10 mg of drug, then diffusion cell was submerged then submerged in a beaker containing 50 mL of PBS (pH 7.4), and incubated in a shaking water bath (Wise bath made by DAIHAN scientific Co. Ltd, Korea) at 37.0 ± 0.5 °C at 50 rpm compared to F1 solution containing same amount of drug (Devaraj et al., 2002; Glavas-Dodov et al., 2002). At suitable time intervals (1, 2, 3, 4, 5, 6, 24, 48, and 72 h), samples were withdrawn and replaced by the same volume of freshly prepared buffer solution to maintain sink conditions. The drug content of the samples was determined using UV spectrophotometer (UV spectrophotometer Jasco v-630 made by Jasco, Tokyo, Japan) at 276 nm. All experiments were done in triplicates (Gürsoy et al., 2004). The obtained data were studied and kinetically analyzed using, Korsmeyer–Peppas semi-empirical model (Siepmann & Peppas, 2011).

## 3. *In vitro* antimicrobial and antibiofilm studies

### 3.1. Antimicrobial evaluation using disc agar diffusion method

Antimicrobial activity of F1, F2, and F3 were determined using disc diffusion method. A sterile cotton swab soaked into *E. faecalis* strain (ATCC 29212) corresponding to a 0.5 McFarland standard solution was used to inoculate the surface of a brain heart infusion agar plate. F1, F2, F3, and CS were tested on agar media by applying amount of each formula that is equivalent to 5 μg of the drug. Saline disc served as negative control. Plates were incubated at 37 °C for 24 h and then the zones of inhibition were measured, experiments were performed in triplicates (Blanscet et al., 2008).

### 3.2. Biofilm inhibition assay

*Enterococcus faecalis* strain (ATCC 29212) was grown overnight in tryptone soy broth with 1% glucose (TSB) at 37 °C. The assay was performed in 96-well flat bottom plates, first six wells in the plate served as control; first three wells had the culture media (TSB) alone, the next three wells had the culture media with *E. faecalis* adjusted to (0.5 Mcfarland) without adding the drug. A volume of 100 μl of culture media with *E. faecalis* adjusted to (0.5 Mcfarland) were added to the rest of the wells. All plates were incubated at 37 °C for 24 h for biofilm formation. The plate was washed with 200 μl saline, then 100 μl of F1, F2, and F3 prepared in a range of drug concentration (15–500 μg/ml) were added to each well. All plates were incubated at 37 °C for 24 h. The content of each well was removed, and washed twice with sterile 200 μl saline after that, the plates were allowed to dry. Biofilms were stained with 100 μl of 0.1% crystal violet for 15 min then washed with 200 μl saline to discard the excess dye. A volume of 200 μl of ethanol was added in each well to remove the dye taken up by the biofilm and the absorbance readings were measured at 600 nm using a microplate reader (ELX 800 made by Biotek, Winooski, VT). The difference between the absorbance of the treated and non-treated biofilm was used to calculate the percentage reduction of biofilm mass, using Equation (2): (%) reduction of biofilm = ((total biofilm control-treated biofilm)/total biofilm control) *100 (Lemos et al., 2010; Sabrah et al., 2013; Zhang et al., 2013; Ong et al., 2017).

### 3.3. Inhibition and formation of biofilm on teeth

The current study was approved by the Ethical Committee of Faculty of Pharmacy, Cairo University. Twelve extracted single rooted teeth free from cracks were selected from Dentistry Hospital in the British University of Egypt. The teeth were autoclaved at 121 °C for 20 min. The sterilized teeth were immersed in 10 ml brain heart infusion broth with 5% glucose containing the same *E. faecalis* strain adjusted to (0.5 McFarland) for 24 h. After biofilm formation, F1, F2, and F3 were added for 24 h in three concentrations 125 μg/ml, 250 μg/ml, and 500 μg/ml, respectively. All teeth were removed and dispersed in sterile saline solution by vortexing (ZX3 Advanced Vortex Mixer by Velp Scientifica made in Italy) to ensure that all biofilm fell off the teeth. The solution was cultured using a sterile swab on brain heart infusion agar plates and the bacterial colonies were counted and recorded as CFU (Steiner-Oliveira et al., 2007; Lemos et al., 2010; Costa et al., 2018; Ewell 1890).

## 4. Statistical analysis

Statistical analysis was performed on *in vitro* biofilm inhibition using IBM SPSS Statistics Version 2.0 for Windows (Chicago, IL). Data were presented as mean and standard deviation (SD). The significance level was set at *p* ≤ .05. Kolmogorov–Smirnov and Shapiro–Wilk tests were used to assess data normality. One-way ANOVA followed by Tukey’s post-hoc test was performed to compare the effect of different dilutions within each nanoparticle type on biofilm inhibition and zone of inhibition. Independent Student-t test was used to compare the effect of both nanoparticles within each dilution on biofilm inhibition and zone of inhibition.

## 5. Results and discussion

### 5.1. Determination of particle size, size distribution, and zeta potential

**Figure 1.**
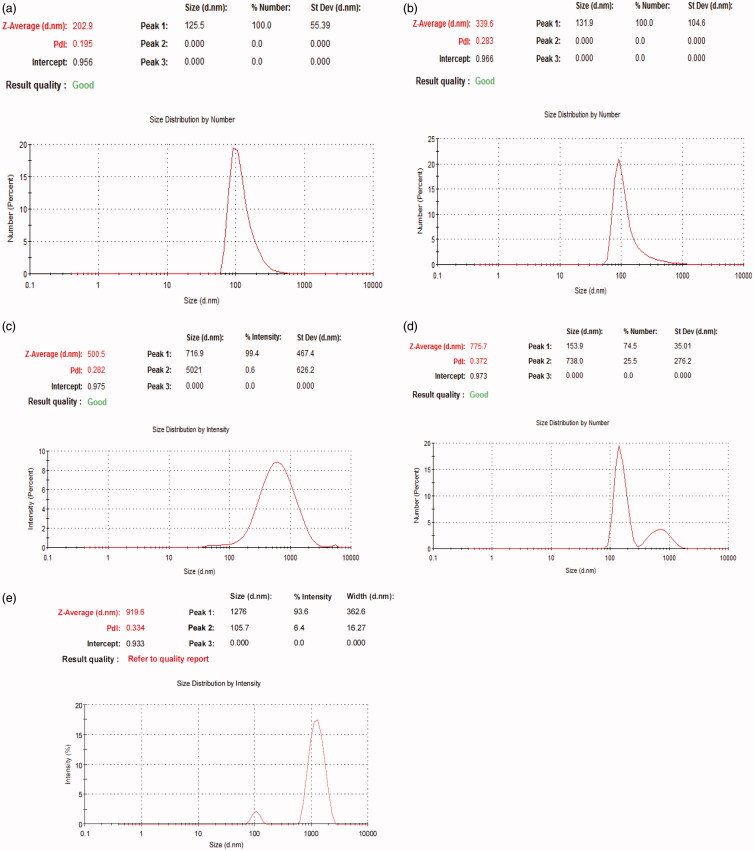
Particle size of (a) F2, (b) F3, (c) F4, (d) F5, and (e) F6.

**Figure 2.**
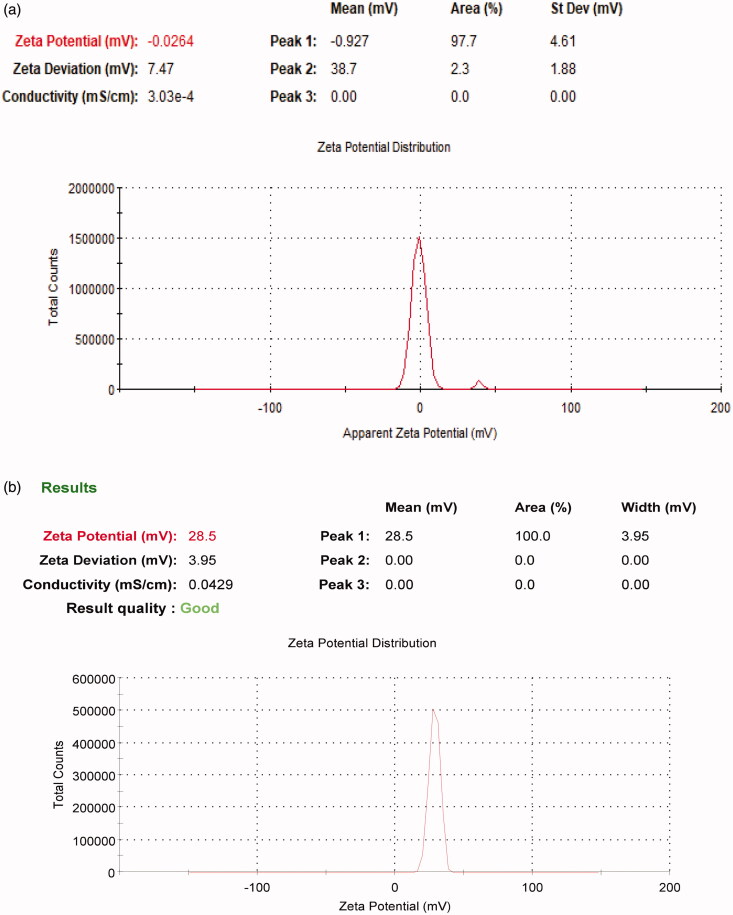
Zeta potential of (a) F2 and (b) F3.

Particle size and zeta potential are two important physiochemical parameters in assessing the nanoparticles. The particle size of the formula of choice should be less than 500 nm to be able to reach dentinal tubules branches (Mjör & Nordahl, 1996). To overcome other ciprofloxacin particles coated with chitosan that were in microns (Jeong et al., 2008a) the conditions of the preparation method were modified to increase sonication time in order to decrease particle size (Dillen et al., 2004; Antoniou et al., 2015). All formulae showed a polydispersity index PDI ranging from 0.195 to 0.372 suggesting that they are monodispersed. All prepared formulas sizes are shown in Table 2 and Figure 1, results revealed that as we increase the concentration of chitosan particle size of nanoparticles increases. Therefore, all formulas were rejected except for F3 which was chosen to be the formula of choice when compared to F2. The average size of F2 is 202.9 nm ± 4.56 nm and PDI is 0.195 ± 0.051, while F3 is 339.6 nm ± 9.8 nm and PDI is 0.283 ± 0.0404. Zeta potential for F2 is −0.0254 ± 0.01mv, while F3 is +28.5 ± 2.5mv which proves the successful coating of PLGA nanoparticles with chitosan as shown in Figure 2.

**Table 2.** Particle size and PDI of prepared formulas (mean ± SD, *n* = 3).

Formula	Size	PDI
F2	202.9 nm ± 4.56nm	0.195 ± 0.051
F3	339.6 nm ± 9.8nm	0.283 ± 0.0404
F4	500.5 nm ± 11.23nm	0.282 ± 0.208
F5	775.7 nm ± 20.4 nm	0.372 ± 0.024
F6	919.6 nm ± 18.9nm	0.334 ± 0.220

### 5.2. Determination of drug encapsulation efficiency

The mean encapsulation efficiency of F2 is 64%±2% while F3 is 78%±5%. This could be explained by two theories; the first was by Dillen et al. (2004) who stated that as we increase the homogenization cycles the EE% increases from 62% till 80%. While, the second theory was based on the concentration of chitosan as explained by Pandit et al. (2017) who stated that EE% for chitosan-coated PLGA nanoparticles of bevacizumab ranged from 50% to 70%, that was higher than PLGA bevacizumab alone.

### 5.3. Differential scanning calorimetry (DSC) analysis

**Figure 3.**
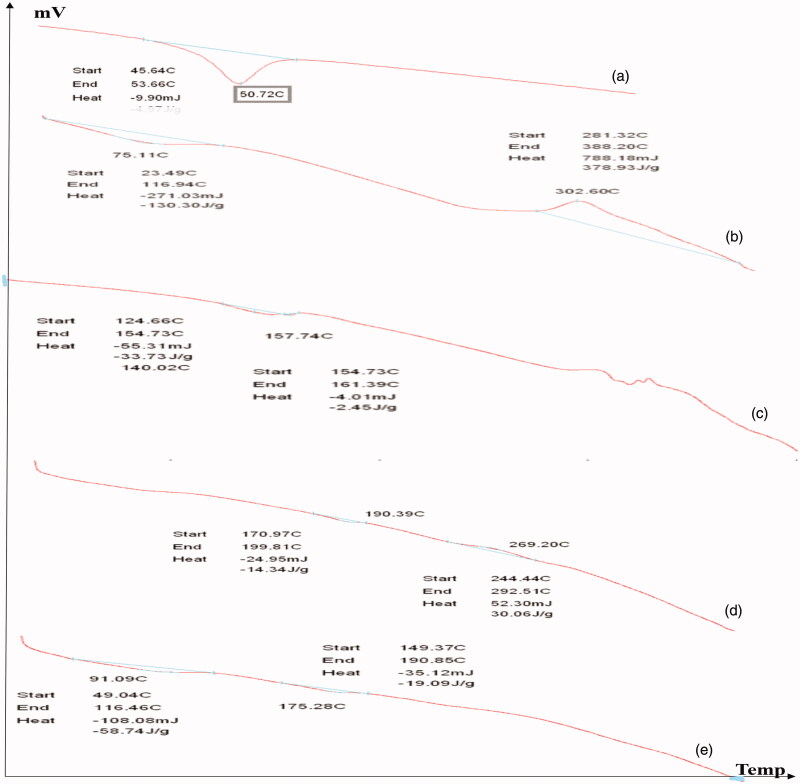
DSC of (a) PLGA, (b) CS, (c) CIP, (d) F2, and (e) F3.

DSC is a thermal analytical technique which provides information about the physical properties of the materials and nanoparticles. Quantitative information about exothermic, endothermic and heat capacity changes of PLGA, CS, CIP, F2, and F3 in terms of temperature were shown in Figure 3. It was demonstrated that PLGA polymer has an endothermic peak at 50.72 °C, the result is in agreement with that obtained by in agreement with Dillen et al. (2004) and Mohammadi et al.. CS has an endothermic peak at 75.11 °C and an exothermic peak at 302.6 °C the results are in agreement with those obtained by Sobhani et al. (2017) and Ali et al. (2011) who stated that the exothermic peak of CS was around 300 °C. The drug alone showed an endothermic peak at 159.96 °C this was in agreement with Dillen et al. (2004) and Sobhani et al. (2017). The F2 nanoparticles shows an endothermic peak 190.39 °C and an exothermic peak at 269.20 °C while the drug endothermic peak disappeared this is in agreement with Dillen et al. (2004) who stated that the endothermic peak of ciprofloxacin in amorphous state could not be measured. The F3 nanoparticles shows two shifted endothermic peaks at 91.09 °C and 175.28 °C this is in consistence with Sobhani et al. who previously studied ciprofloxacin and chitosan nanoparticles and showed two endothermic peaks at 169.1 °C and 212 °C. DSC analysis did not show interactions between ciprofloxacin and PLGA or Chitosan. This also, proves drug encapsulation in the introduced nanoparticles.

### 5.4. X-ray diffraction (XRD)

**Figure 4.**
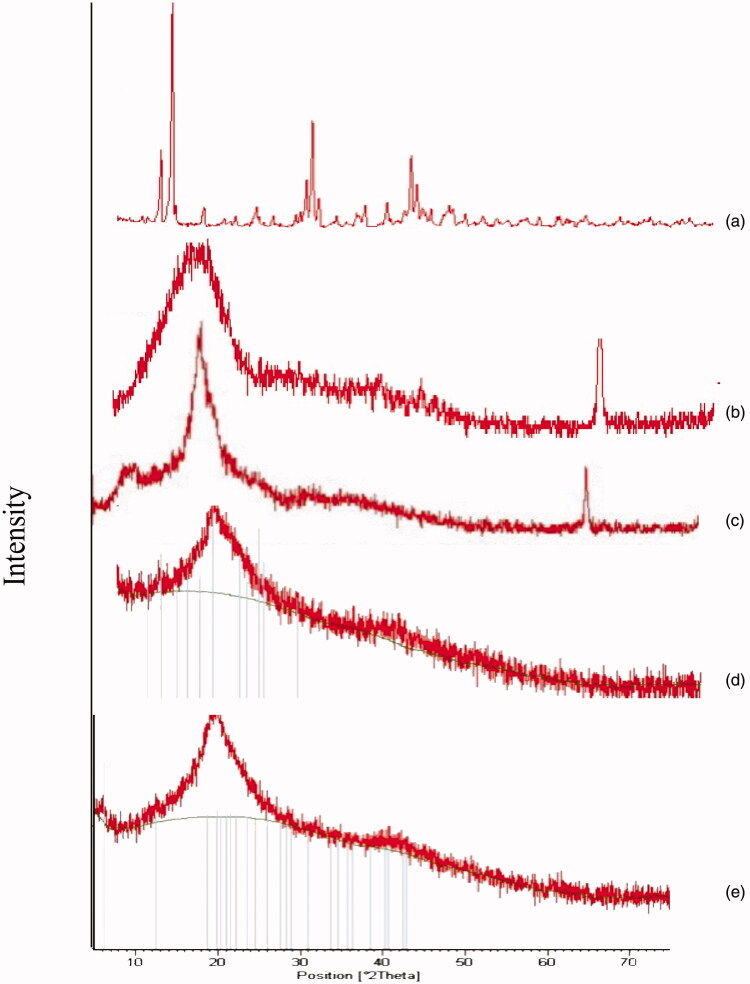
XRD of (a) CIP, (b) PLGA, (c) CS, (d) F2, and (e) F3.

The XRD pattern is shown in Figure 4 reveals that PLGA and CS are amorphous polymers the results were in consistence with those obtained by Kang et al., Makadia et al., and Okano et al. (Dillen et al., 2004; Okano et al., 2009; Makadia & Siegel, 2011; Kang et al., 2017) who stated that both polymers are amorphous, while CIP is crystalline drug as previously reported by Dillen et al. (2004). In conclusion, the disappearance of the characteristic peak of the drug because drug is fully encapsulated and coated with the polymers, or may be converted into the amorphous state, this was in consistence with Dillen et al. and Mohammadi et al. (Dillen et al., 2004; Mohammadi et al., 2010; Kang et al., 2017).

### 5.5. Fourier transforms infrared (FTIR) spectroscopy

**Figure 5.**
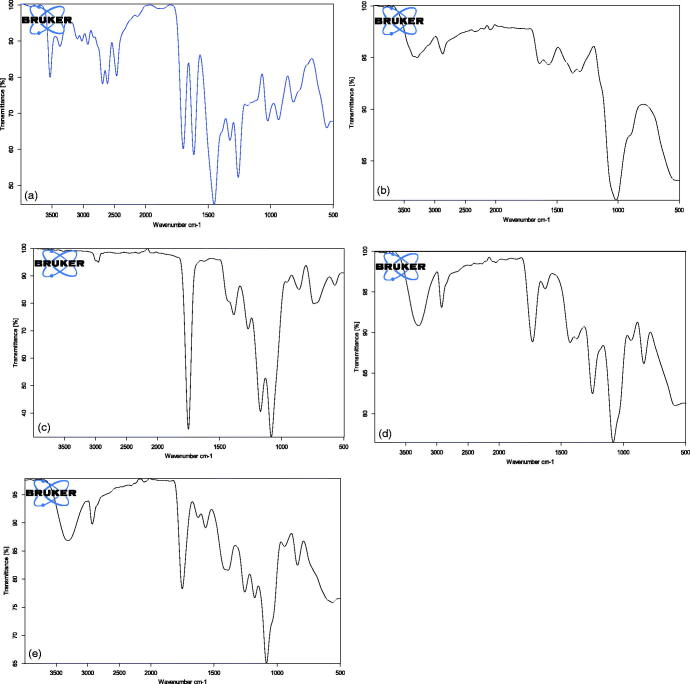
FT-IR (a) CIP, (b) CS, (c) PLGA, (d) F2, and (e) F3.

Figure 5 shows the FTIR spectra for PLGA, CS, CIP, F2, and F3. CIP shows a characteristic band of OH group between 3450 and 3500 cm^−1^ known as OH stretching vibration. The bands at 3000 and 2950 cm^−1^ represent the alkenes and aromatic C–H stretching. The band at 1700–1750 cm^−1^ indicated carbonyl C=O stretching. The peak between 1600 and 1650 cm^−1^ was assigned to quinolones. The band at the 1400–1450 cm^−1^ represented C–O and the peaks at 1250–1300 cm^−1^ suggested bending vibration of the O–H group which indicated the presence of carboxylic acid. In addition, a strong absorption peak between 1000 and 1050 cm^−1^ was assigned to the C–F group (Tom et al., 2004; Sahoo et al., 2011). CS characteristic peaks are amide groups presence of the residual N-acetyl group were confirmed by the bands at around 1650 cm^−1^ (C=O stretching of amide I) and 1350 cm^−1^ (C–N stretching of amide III), respectively. An absorption band at 3450 cm^−1^ indicates O–H stretching, however, the band at 2880 cm^−1^ indicates CH-stretching. The major absorption band between 1020 and 1090 cm^−1^ represents the anti-symmetric stretching of the C–O–C bridge, however, the skeletal vibrations involving the C–O stretching are characteristic of its saccharide structure (Povea et al., 2011; Antonino et al., 2017). PLGA shows bands of symmetric and asymmetric stretching of CH2 and CH3. The bands of the symmetric stretching are present between 2850 and 2980 cm^−1,^ while the band of asymmetric stretching for CH3 is 1375 cm^−1^and CH2 is 1450 cm^−1^. The band at 1760 cm^−1^ caused by the C=O bond stretching of the esters (Yildiz et al., 2018). The 1110 cm^−1^ and the 1185 cm^−1^ are found to be bands relative to the C–O stretching of aliphatic polyesters this is in consistence with Yildiz et al. (2018) who stated that alkyl C – H stretch peak at 2950–2850 cm^−1^, ester C=O stretch peak at 1750–1735 cm^−1^, and C – O–C stretch peak at 1250–1050 cm^−1^, respectively. The drug spectra in F2 and F3 shows the attenuated peaks of the drug, the main characteristic peaks are a broad peak between 3400 and 3500 cm^−1^ indicates OH stretching vibration (intermolecular hydrogen bonding) and the peaks between 1600 and 1650 cm^−1^ was assigned to quinolones and they are present in F2 and F3, this is in agreement with many reports (Tom et al., 2004; Sahoo et al., 2011). Finally, the band at 1760 cm^−1^ for C=O bond stretching of the esters is combined with the peak of quinolones in F2 and F3. In F2 and F3 the C–O stretching peak of PLGA are shifted to 1120 cm^−1^ and 1250 cm^−1^ instead of 1110 cm^−1^ and the 1185 cm^−1^, this is maybe due to interaction between the polymer and the drug. The results are in agreement with those obtained by Farajzadeh et al. (2018) who stated that peaks of PLGA are shifted due to interaction with curcumin. In F3 the attenuated peaks of chitosan at 1650 cm^−1^ and 1350 cm^−1^ of the (amide I and III stretching) appeared with no interaction with the drug in the nanoparticles. Also, the peak between 1020 and 1090 cm^−1^ is shifted to 1100 cm^−1^ to be combined with the peak of PLGA. No clear shift in position of absorption bands of ciprofloxacin hydrochloride in F3 this indicates that there is no significant interaction between the drug and the polymers in the nanoparticles (Ibrahim et al., 2015).

### 5.6. Scanning and transmission electron microscope

**Figure 6.**
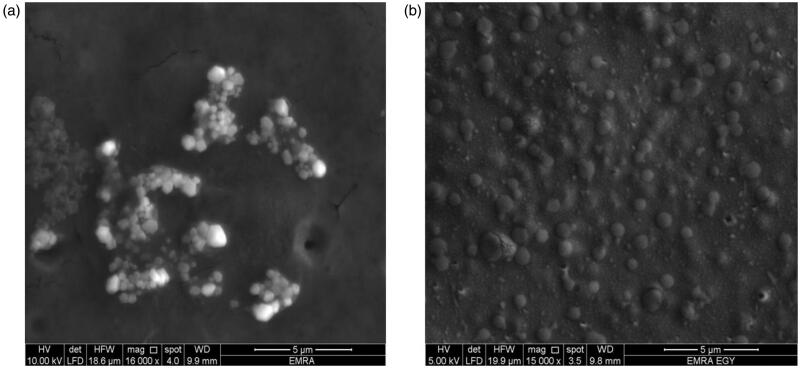
SEM images of the nanoparticles (a) F2 and (b) F3.

**Figure 7.**
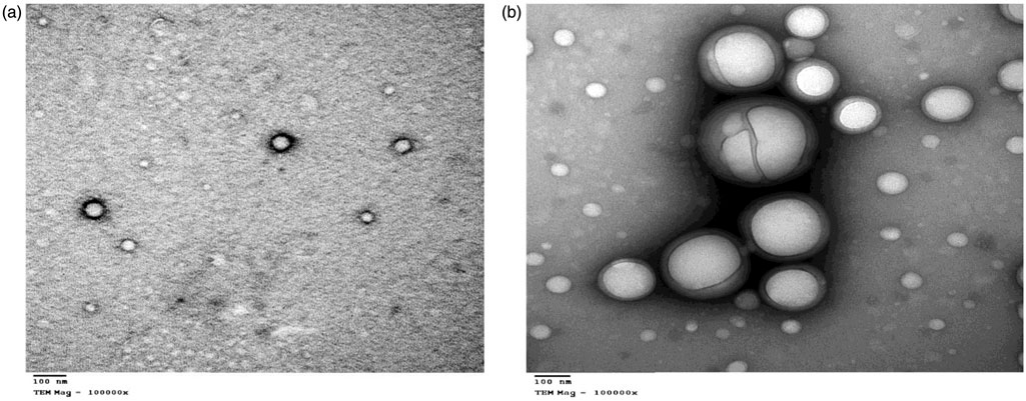
TEM images of the nanoparticles (a) F2 and (b) F3.

The SEM images below show spherical shape of nanoparticles for both F2 and F3 as shown in Figure 6 also, F3 shows no particle aggregation. The TEM image of F2 and F3 are spherical nanoparticles this is shown in Figure 7.

### 5.7. *In vitro* drug release

**Figure 8.**
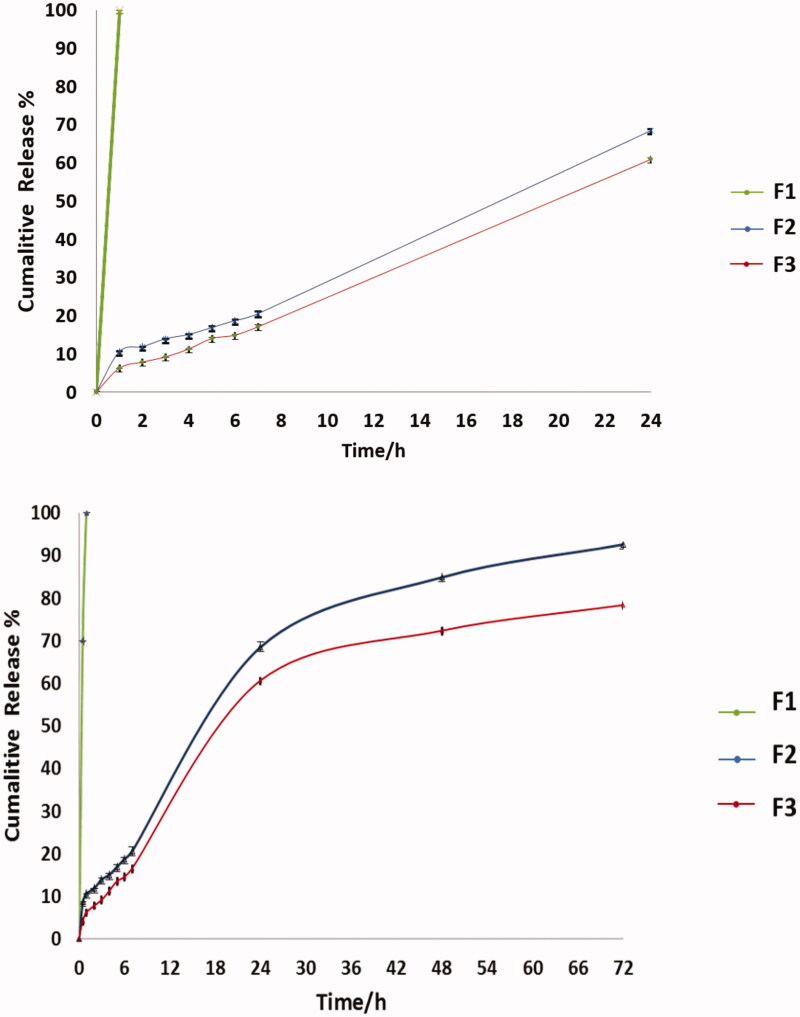
Cumulative drug release percent of F1, F2, and F3 (mean ± SD, *n* = 3).

The cumulative percent release of CIP from the nanoparticles F2 and F3 compared to F1 as a control are shown in Figure 8. The F1 was 100% released in the first hour. The release of the drug in the first 24 hours was 68.5% and 60.57% in F2 and F3, respectively. The cumulative % release after 72 hours was 92.62% for F2 this was in agreement with Jeong et al. (2008b) and Song et al. (2008) who stated that around 60% of drug is released in the first 24 h from PLGA followed by slow release over days. The release of drug in F3 is 78.3%, as noted it was slower than F2 due to coating with chitosan, as reported in previous studies which stated that chitosan coat hinder the release of the drug, also charge attraction between PLGA and chitosan played an important role in sustained drug release (Chen et al., 2016; Al-Nemrawi et al., 2018; Lu et al., 2019). The kinetics revealed that the in vitro release of F2 is zero-order kinetics, as the plots showed the maximum linearity (*R*^2^ = 0.951), in this case the rate of drug release is independent of its dissolved concentration in the release medium and is delivered at a constant rate over time (Majumder et al., 2018). Meanwhile, the *in vitro* release of F3 was best explained by Higuchi diffusion model order kinetics as the plots showed the primary linearity (*R*^2^ = 0.981), the Higuchi model describes, purely diffusion-controlled drug release from the polymers (Kariminia et al., 2016; Pandit et al., 2017). The *R*^2^ values are listed in Table 3.

**Table 3.** The *R*^2^ values of F2 and F3.

Formula	Zero Order	First order	Higuchi diffusion model
F2	0.951	0.935	0.903
F3	0.961	0.979	0.981

### 5.8. Antimicrobial activity using disc agar diffusion method

As *E. faecalis* is found in most of root canal infections (Rôças et al., 2004), Figure 9 shows the plates with zones of inhibition around the medicated discs. Antimicrobial activities of F1, F2, F3, and CS were studied after 24 h of incubation at 37 °C. Results revealed that F3 was significantly more effective against *E. faecalis* than CS, F2, and F1with *p* ≤ .05. The mean diameter of inhibition zones observed against *E. faecalis* were 12 ± 0.1 mm, 22 ± 0.5 mm, 20 ± 0.3 mm, and 32 ± 0.5 mm for CS, F1, F2, and F3, respectively; where CS> (F1 and F2), also (CS and F1)> F2, finally F3> CS, F1 and F2. These results proved that F3 exhibited higher antimicrobial activity due to the combined effect of chitosan with the ciprofloxacin. This proves that ciprofloxacin with chitosan has better antimicrobial activity than the antibiotic alone, this agreed with Sobhani et al., who stated that CIP-CS nanoparticles decreased Minimum inhibitory concentration of gram positive and gram negative bacteria with 50% (Kishen et al., 2008; Nguyen et al., 2017; Sobhani et al., 2017). The higher the antibacterial activity of nanoparticles F3 may be due to interaction with the bacterial cell membrane, due to the positive charge of chitosan that adheres to the negative charge of the bacterial cell membrane.

**Figure 9.**
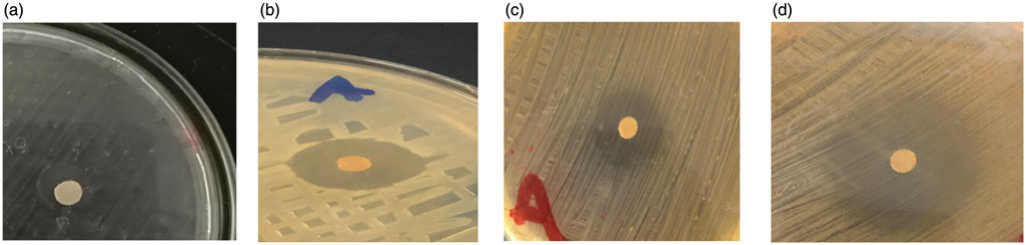
Antimicrobial effect of (a) CS, (b) F1, (c) F2, and (d) F3.

### 5.9. Biofilm inhibition assay

**Figure 10.**
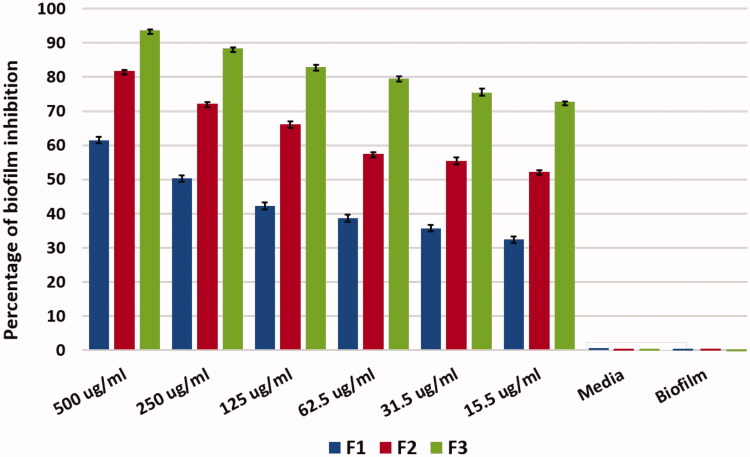
Statistical analysis of biofilm inhibition assay (mean ± SD, *n* = 3).

Formation of biofilm was proved using plate reader that the biofilm was formed as 100% in the positive control and the media alone as a negative control showed no reading and no biofilm. The results show that at 500 μg/ml F1 eradicated biofilm with 60% where the F2 showed eradication of biofilm with 74% and F3 eradicated biofilm by 91.8%. Therefore, the use of chitosan with antibiotic in Nano-form is more effective on biofilm eradication than the antibiotic alone with 32% and the antibiotic in Nano-form with 18%. There was gradual decrease in biofilm eradication as we decrease the drug concentration and also F3 was more effective in biofilm eradication more than F1and F2. This result shows a significant difference with *p* value ≤.05 where F3 shows antibiofilm activity over F1 and F2, this gives F3 formula privilege in eradicating the biofilm as shown in Figure 10. Biofilm eradication is quite difficult; however, using antibiotic alone is not quite effective due to bacterial resistance and biofilm formation. As a result, nanotechnology and nanoparticles are now under application for biofilm eradication (Kishen & Shrestha, 2015). Nanoparticles coated with chitosan showed that eradication of biofilm is applicable and this was in agreement with Ong et al., Kishen et al., and Shrestha et al. (Kishen et al., 2008; Shrestha et al., 2010; Ong et al., 2017) they stated that using chitosan nanoparticles reduce biofilm and disrupt its structure. This reveals that nanoparticles are able to penetrate biofilm and eradicate bacteria (Avadi et al., 2004; Nguyen et al., 2017).

### 6. Inhibition and formation of biofilm on teeth

The biofilm of *E. faecalis* was abolished markedly, and the number of viable bacterial cells in the biofilms was reduced significantly using F3 in three different concentrations 125, 250, and 500 μg/ml, respectively. This antibiofilm efficacy improved when the concentration of F1, F2, and F3 increased. The results of F1, F2, and F3 CFU are shown in Table 4. The results revealed that there is a significant difference between F3 with chitosan coat and the F2 without chitosan; where F3 is way more effective on biofilm inhibition. Possible explanations for these observations would be the interaction between positively charged Chitosan and negatively charged bacterial cells during the 24 h resulting in most of the bacteria were inhibited. Results showed a marked decrease in the bacterial count (Liu et al., 2001; Pagonis et al., 2010) with the formula that contains chitosan F3. Chitosan has proven to have an antibiofilm effect (Kishen et al., 2008; Nair et al., 2018).

**Table 4.** CFU of F1, F2, and F3 with different concentrations (mean ± SD, *n* = 3).

Concentration/Formula	F1	F2	F3
500 μg/ml	225CFU ± 12.3	144CFU ± 19	72CFU ± 2.6
250 μg/ml	>300 CFU	286CFU ± 21.2	103CFU ± 5.0
125 μg/ml	>300 CFU	>300 CFU	227 CFU ± 5.9

## 7. Conclusion

Formulation of ciprofloxacin nanoparticles with PLGA and coating it with chitosan were prepared, all the prepared nanoparticles showed controlled drug release pattern, also the coated nanoparticles with cationic chitosan exhibited higher encapsulation efficiency, higher inhibition zone and higher antibiofilm effect than the antibiotic alone and the nanoparticles without Chitosan. Chitosan exhibited antimicrobial and antibiofilm effect on *E. fecalis,* consequently the prepared nanoparticles would be of great interest in treating root canal infected patients using chitosan coated PLGA nanoparticles entrapping Ciprofloxacin as new delivery system with controlled drug release.
